# Domain-Specific Physical Activity and Stroke in Sweden

**DOI:** 10.1001/jamanetworkopen.2024.13453

**Published:** 2024-05-29

**Authors:** Adam Viktorisson, Annie Palstam, Fredrik Nyberg, Christina Berg, Lauren Lissner, Katharina S. Sunnerhagen

**Affiliations:** 1 Department of Clinical Neuroscience, Institute of Neuroscience and Physiology, Sahlgrenska Academy, University of Gothenburg, Gothenburg, Sweden; 2 Department of Rehabilitation Medicine, Sahlgrenska University Hospital, Gothenburg, Sweden; 3 School of Health and Welfare, Dalarna University, Falun, Sweden; 4 School of Public Health and Community Medicine, Institute of Medicine, Sahlgrenska Academy, University of Gothenburg, Gothenburg, Sweden; 5 Department of Food and Nutrition, and Sport Science, University of Gothenburg, Gothenburg, Sweden

## Abstract

**Importance:**

Associations of domain-specific physical activity with stroke incidence and poststroke outcomes have not been extensively studied using long-term, population-based data.

**Objective:**

To investigate associations of leisure time, work time, transport, and household physical activity with stroke incidence and death or dependency in activities of daily living (ADL) 3 months after stroke.

**Design, Setting, and Participants:**

The prospective, population-based Interplay Between Genetic Susceptibility and External Factors (INTERGENE) cohort study was conducted among a random sample of individuals from an urban-rural area covering western Sweden; 3614 individuals aged 24 to 77 years were examined in 2001 to 2004, and 1394 individuals were reexamined in 2014 to 2016. The median (range) follow-up was 20.0 years (56 days to 21.9 years). Data were analyzed from September through October 2023.

**Exposure:**

Physical activity levels were self-reported for leisure time, work time, transportation, and household domains. The mean number of steps taken over a 6-day period was collected in a subgroup of participants using a sealed pedometer.

**Main Outcomes and Measures:**

Follow-up for stroke incidence and mortality rates continued until December 31, 2022. The composite outcome of death or ADL dependency was assessed at 3 months after stroke.

**Results:**

Among 3614 individuals (mean [SD] age, 51.4 [13.1] years; 1910 female [52.9%]); 269 individuals (7.4%) developed stroke, of whom 120 individuals (44.6%) were dead or ADL dependent at 3 months. Intermediate (adjusted hazard ratio [aHR], 0.54; 95% CI, 0.38-0.77) and high (aHR, 0.47; 95% CI, 0.31-0.73) levels of leisure time physical activity were associated with a reduced incidence of stroke compared with low levels, as was an intermediate level of physical activity in transportation (aHR, 0.69; 95% CI, 0.52-0.93). High levels of leisure time physical activity were also associated with a reduced risk of poststroke death or ADL dependency (adjusted odds ratio, 0.34; 95% CI, 0.16-0.71) compared with low levels. Work time and household physical activity were not associated with stroke incidence or stroke outcomes. In exploratory subgroup analyses, there were interactions between physical activity and smoking (current smoking or smoking in the past year associated with stroke risk only in participants with low or intermediate physical activity: aHR, 2.33; 95% CI, 1.72-3.15) and family history of stroke (first-degree relative with a history of stroke associated with stroke risk only in participants with low or intermediate physical activity: aHR, 1.73; 95% CI, 1.27-2.38).

**Conclusions and Relevance:**

In this study, leisure time and transport-related physical activities were associated with a reduced risk of stroke. A high level of leisure time physical activity was also associated with a lower risk of death or ADL dependency 3 months after stroke.

## Key Points

**Question:**

Are levels of domain-specific physical activity associated with stroke incidence and stroke outcomes in the general population?

**Findings:**

In this cohort study with data obtained from 3614 participants, higher levels of leisure time and transport physical activity were associated with a reduced stroke risk, while high levels of leisure time physical activity were associated with a lower risk of death and dependency in activities of daily living 3 months after stroke.

**Meaning:**

These findings suggest that integrating leisure time physical activity and active transportation into public health policies may be a sustainable intervention to reduce the burden of stroke.

## Teaser

This cohort study investigates the association of leisure time, work time, transport, and household physical activity with stroke incidence and outcomes in Sweden.

## Introduction

Public strategies that promote physical activity may effectively reduce the burden of cardiovascular diseases.^1^ Objective measurements indicate a dose-response association between physical activity and stroke-free survival,^2,3^ and modifiable factors, including physical inactivity, have been associated with a substantial proportion of the population-attributable stroke risk in all major regions of the world.^4^ Increasing physical activity is a sustainable concept that can improve overall health,^5^ prevent other cardiovascular risk factors,^6^ and stimulate several potentially neuroprotective mechanisms.^7^

Although previous research has highlighted the beneficial association of overall physical activity with reduced risk of stroke, many details concerning the association between prestroke physical activity and stroke outcomes remain unclear.^8^ Most prior studies have not distinguished domain-specific physical activity, including leisure time, work time, transport, and household physical activity, when estimating the risk of stroke and poststroke outcomes.^9^ While leisure -time physical activity has consistently been associated with a reduced risk of stroke,^10,11,12^ studies have yielded conflicting results regarding work time physical activity, indicating both a decreased risk^13^ and an increased risk.^14^ Additionally, there is a lack of data on the associations of transport or household physical activity with stroke. Device-based measurements fail to capture information about physical activity domains, potentially favoring dynamic activities, such as walking or running, over resistance activities, such as weight lifting or isometric exercises. Conversely, self-reported measures are susceptible to social desirability and recall biases.^15^

A better understanding of domain-specific associations of physical activity is crucial for tailoring stroke-prevention strategies. Moreover, prospective, population-based data on the association between domain-specific physical activities and stroke are scarce. In this study, we aimed to investigate associations of domain-specific physical activity with stroke incidence and poststroke death or activities of daily living (ADL) dependency using a prospective, population-based cohort.

## Methods

This prospective cohort study was based on data from a randomly sampled population cohort established by the Interplay Between Genetic Susceptibility and External Factors (INTERGENE) research program in 2001.^16^ Data collection for the INTERGENE cohort was approved by the Regional Ethics Committee in Gothenburg. Data linkage to national registries for this study was approved by the Swedish Ethical Review Authority. All participants provided written informed consent to take part in examinations, including linkage to future health end points. This study follows the Strengthening the Reporting of Observational Studies in Epidemiology (STROBE) reporting guideline for cohort studies.

An invitation to participate was sent out to 8625 peopled aged 24 to 74 years living in an urban-rural area covering western Sweden. The setting comprised Sweden’s second-largest city, Gothenburg (urban), and smaller communities and dispersed settlement areas throughout the region (rural). Baseline data, including questionnaires and clinical measurements, from 3614 participants (41.9% acceptance rate) were collected from 2001 to 2004. Baseline assessments were performed at the Sahlgrenska University Hospital in Gothenburg and using a mobile research bus. Reexaminations using the same metrics were performed from 2014 to 2016 in 1394 participants (of 2108 invited) who attended the baseline examination at Sahlgrenska University Hospital and were still alive.

### Physical Activity Assessments

Self-reported physical activity levels were evaluated using questionnaires administered on the day of examination across 4 domains: leisure time, work time, transportation, and household physical activity. Participants were asked to estimate their mean physical activity level over the past year and consider significant variations that may have occurred between seasons. Leisure time physical activity was evaluated using the validated Saltin-Grimby Physical Activity Level Scale.^17,18^ Work time, transportation, and household physical activity questions were part of a validated, self-administered physical activity questionnaire that was used in the Swedish National Public Health Survey.^19^ For each domain, physical activity levels were categorized as low, intermediate, or high (eTable 1 in Supplement 1). These assessments were repeated during reexaminations.

Objective evaluations of physical activity were performed in a subgroup of 496 participants at reexaminations using a sealed pedometer: Yamax CW 700 (Yamax Health and Sports). The Yamax pedometer has been found to have high step count accuracy when worn on the hip.^20^ Participants were instructed by a research nurse to wear the pedometer on the hip during all waking hours for 6 days. To account for activity during transportation, 160 steps were added to the total step count per reported minute of cycling. The mean daily step count was calculated for participants who had recorded at least 1000 steps per day over a period of 10 hours for 4 consecutive days. Investigators repeatedly tested the function of all pedometers before and throughout the study.

### Participant Characteristics

Participant characteristics considered as potential risk indicators were obtained at baseline (2001-2004) and categorized as lifestyle factors, socioeconomic factors, comorbid conditions, or genetic factors.^16^ Lifestyle factors included smoking (current smoking or smoking in the past year), high alcohol intake (>10 g/d for females and >20 g/d for males), consumption of red meat more than 5 times per week, consumption of vegetables (including mixed vegetables, carrots, beets, cabbage, cauliflower, broccoli, spinach, and vegetable soups or dishes) more than 5 times per week, and consumption of sweets (including pastries, cookies, cakes, candy, chocolate, or ice cream) more than 5 times per week. Socioeconomic factors included having a postsecondary education (>12 years), financial stability (managing monthly expenses without difficulty), being married or partnered, a social network (communication or engagement with ≥3 familiar individuals per week), and residence in an urban metropolitan area. Comorbid conditions included obesity (body mass index [calculated as weight in kilograms divided by height in meters squared] ≥30), diabetes, hyperlipidemia, hypertension, and atrial fibrillation, which were self-reported or identified via physical examinations performed within this study.^16^ Genetic factors included a first-degree relative (father, mother, or full sibling) with a history of stroke, apolipoprotein E gene allele ε4, and insertions or deletions in the apolipoprotein C1 gene.

### Stroke Incidence and Outcomes

Using Swedish personal identification numbers, data collected in the INTERGENE cohort were linked to data from national Swedish registries. Stroke events were identified in the National Patient Register held by the National Board of Health and Welfare in Sweden using *International Statistical Classification of Diseases and Related Health Problems, Tenth Revision *(*ICD-10*) codes I61, I63, and I64. The use of *ICD-10* in Sweden started in 1997. Additional incident stroke deaths during follow-up were identified in the Swedish Cause of Death Register using the same *ICD-10* codes. First-time stroke events, stroke recurrence, stroke mortality, and all-cause mortality rates were followed up until December 31, 2022.

A severe stroke outcome was defined as a composite measure of death (all-cause mortality) or ADL dependency at 3 months. Poststroke ADL dependency was assessed from a postal questionnaire sent at 3 months to all patients registered with stroke in Sweden via the Swedish Stroke Register (Riksstroke).^21^ ADL dependency was identified if any of the following conditions were met: having reduced indoor mobility, requiring assistance with toileting, needing help with dressing, or relying on a caregiver for all ADL.

### Statistical Analysis

Baseline characteristics were presented as numbers with percentages for categorical variables and means with SDs for continuous variables. Spearman rank correlation was used to test the correlation between self-reported physical activity and pedometer-derived data. Follow-up time was calculated as the time in days from the date of cohort entry until the date of the first stroke diagnosis, death, or end of follow-up, whichever occurred first. There was no loss to follow-up in national registers. However, emigration history was not known. Age-adjusted cumulative stroke hazards stratified by domain-specific physical activity level and accounting for the competing risk of death were calculated using hazard regression models.^22^ Crude cumulative incidences of stroke were also calculated using Kaplan-Meier survival curves.

Multiple imputation by chained equations was used to handle missing observations in regression analyses. We used the classification and regression trees method, which imputes missing values iteratively by constructing estimating models for each variable with missing data, leveraging all other variables in the dataset.^23^ Adjusted hazard ratios (aHRs) for the association between physical activity and first stroke incidence were calculated using Cox proportional hazard regression models. Schoenfeld residuals were used to test the proportional hazard assumption. Adjusted odds ratios for the association between physical activity and death or dependency 3 months after stroke were calculated using binary logistic regression models. For each physical activity domain, we constructed regression models adjusted for age and sex. In addition, a fully adjusted Cox regression model was fitted for each physical activity domain, including all baseline characteristics.

Mixed-effects Cox regression models adjusted for age and sex were used to assess the association between 2 repeated measurements of domain-specific physical activity and stroke incidence (at baseline in 2001-2004 and at reexaminations in 2014-2016). In these analyses, the follow-up was split at the time of reexamination and physical activity was treated as a time-dependent categorical variable. Subgroup analyses adjusted for age and sex were conducted by exploring the interaction between baseline characteristics and leisure time physical activity using Cox proportional hazard models.

All statistical analyses were 2-tailed, and significance was interpreted based on 95% CIs not including 1 or *P* < .05. All analyses were conducted using R statistical software version 4.3.2 (R Project for Statistical Computing). Data were analyzed from September through October 2023.

## Results

There were 3614 participants (aged between 24 and 77 years at baseline; mean [SD] age, 51.4 [13.1] years; 1910 female [52.9%]) (Table 1). The numbers of participants in each physical activity category at baseline and at reexaminations with corresponding pedometer-derived data are presented in eTable 1 in Supplement 1. The mean number of steps recorded over a 6-day period exhibited positive correlations with self-reported leisure time physical activity (*r* = 0.24; *P* < .001) and transport physical activity (*r* = 0.17; *P* < .001). In contrast, there was a negative correlation between the mean number of steps and household physical activity (*r* = −0.11; *P* = .02) and no correlation with work time physical activity (*r* = 0.01; *P* = .86).

**Table 1.** Baseline Characteristics of Study Participants

Characteristic	Participants, No. (%)			Missing, No.
	Overall (N = 3614)	Stroke events during follow-up (n = 269)	Death or ADL dependency at 3 mo after stroke (n = 120)^a^	
Age, mean (SD), y	51.4 (13.1)	62.5 (9.8)	64.4 (9.9)	0
Sex				
Female	1910 (52.9)	118 (43.9)	60 (50.0)	0
Male	1704 (47.1)	151 (56.1)	60 (50.0)	
Physical activity level				
Leisure time				
Low	370 (10.2)	35 (13.0)	17 (14.2)	24
Intermediate	2264 (62.6)	181 (67.3)	89 (74.2)	
High	956 (26.5)	52 (19.3)	14 (11.7)	
Work time				
Low	1078 (29.8)	72 (26.8)	22 (18.3)	28
Intermediate	1252 (34.6)	85 (31.6)	43 (35.8)	
High	1256 (34.8)	110 (40.9)	42 (35.0)	
Transport related				
Low	1194 (33.0)	85 (31.6)	37 (30.8)	42
Intermediate	1499 (41.5)	98 (36.4)	41 (34.2)	
High	879 (24.3)	80 (29.7)	39 (32.5)	
Household				
Low	1113 (30.8)	78 (29.0)	28 (23.3)	31
Intermediate	1636 (45.3)	117 (43.5)	54 (45.0)	
High	834 (23.1)	69 (25.7)	36 (30.0)	
Lifestyle factor				
Smoking^b^	640 (17.7)	66 (24.5)	30 (25.0)	32
High alcohol intake^c^	467 (12.9)	42 (15.6)	19 (15.8)	14
Food consumption >5 times/wk				
Sweets	1389 (38.4)	114 (42.4)	54 (45.0)	15
Vegetables	1031 (28.5)	61 (22.7)	24 (20.0)	17
Red meat	1377 (38.1)	108 (40.1)	51 (42.5)	13
Socioeconomic factor				
Education >12 y	1106 (30.6)	47 (17.5)	36 (30.0)	31
Financial stability^d^	3191 (88.3)	243 (90.3)	19 (15.8)	47
Married or partnered	2708 (74.9)	195 (72.5)	108 (90.0)	29
Social network^e^	3039 (84.1)	213 (79.2)	83 (69.2)	41
Urban metropolitan area	1637 (45.3)	120 (44.6)	53 (44.2)	0
Comorbid condition				
Obesity	644 (17.8)	71 (26.4)	34 (28.3)	1
Diabetes	128 (3.5)	22 (8.2)	12 (10.0)	12
Hyperlipidemia	421 (11.6)	56 (20.8)	26 (21.7)	12
Hypertension	594 (16.4)	93 (34.6)	38 (31.7)	12
Atrial fibrillation	236 (6.5)	22 (8.2)	12 (10.0)	12
Genetic factor				
Family history of stroke	576 (15.9)	62 (23.0)	23 (19.2)	53
APOE ε4	939 (26.0)	75 (27.9)	37 (30.8)	410
APOC1 insertion or deletion	594 (16.4)	94 (34.9)	37 (30.8)	399

Abbreviations: ADL, activities of daily living; APOE ε4, apolipoprotein E gene ε4 allele; APOC1, apolipoprotein C1 gene.

^a^ There were 54 participants with incomplete ADL assessments.

^b^ Smoking was defined as current smoking or smoking in the past year.

^c^ High alcohol intake was defined as more than 10 g/d for females and more than 20 g/d for males.

^d^ Financial stability was defined as managing monthly expenses without difficulty.

^e^ Presence of a social network was defined as communication or engagement with 3 or more familiar individuals per week.

During a median (range) follow-up of 20.0 years (56 days to 21.9 years), 269 participants (7.4%) experienced a stroke, among whom 115 individuals (42.8%) also had 1 or more recurrent strokes. Age-adjusted cumulative stroke hazards stratified by domain-specific physical activity levels are presented in the Figure. Domain-specific crude cumulative stroke incidences are presented in eFigure 1 in Supplement 1. Among first-incident strokes, there were 207 ischemic strokes (77.0%), 33 intracerebral hemorrhages (12.3%), and 24 stroke deaths with a nonspecified etiology (10.8%). Of 677 deaths during the follow-up, 63 deaths (9.3%) were caused by stroke. At 3 months after stroke, 120 participants (44.6%) were dead or ADL dependent.

**Figure.**
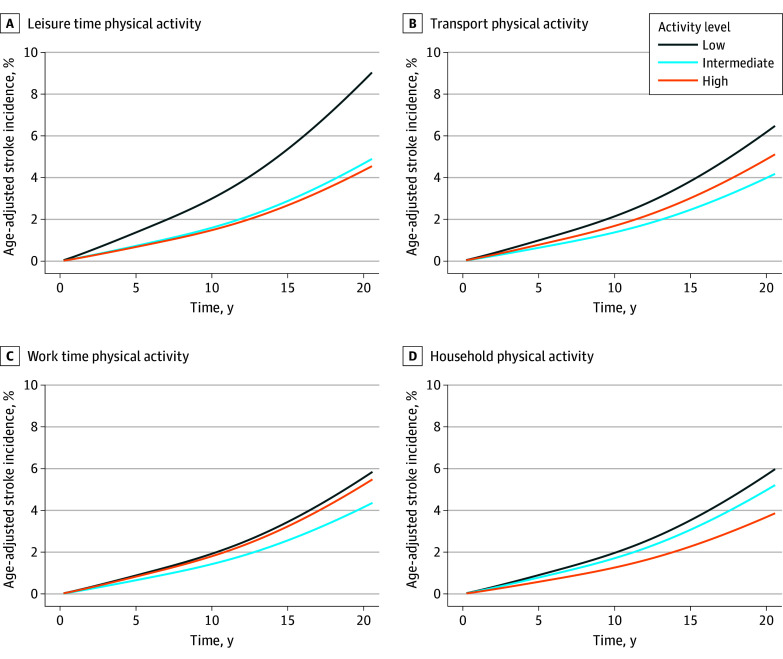
Age-Adjusted Cumulative Incidences of Stroke Over 20-Year Follow-Up Age-adjusted cumulative incidences of stroke were calculated over a 20-year follow-up, stratified by domain-specific physical activity and accounting for the competing risk of death. For each level of physical activity, the model estimated the 1 − survival probability over time for a person at the mean age of the study population.

Adjusted associations of domain-specific physical activity levels with stroke incidence and poststroke death or ADL dependency are presented in Table 2. Models adjusted separately for lifestyle factors, socioeconomic factors, comorbid conditions, and genetic factors are presented in eTable 2 in Supplement 1. Intermediate and high levels of leisure time physical activity were associated with a decreased incidence of stroke compared with low levels across all models; for example, aHRs in model 1 were 0.54 (95% CI, 0.38-0.77) for intermediate and (aHR, 0.47; 95% CI, 0.31-0.73) for high levels. An intermediate compared with a low level of physical activity in transportation was also associated with a reduced stroke hazard in all models (eg, model 1: aHR, 0.69; 95% CI, 0.52-0.93). However, there was no association for a high level of transport physical activity. Work time and household physical activity showed no association with stroke incidence. A high compared with a low level of leisure time physical activity level at baseline was associated with a lower risk of death or ADL dependency 3 months after stroke in all models (eg, model 1: adjusted odds ratio, 0.34; 95% CI, 0.16-0.71).

**Table 2.** Associations of Baseline Domain-Specific Physical Activity With Stroke Incidence and Outcomes^a^

Physical activity type^b^	First stroke, aHR (95% CI)^c^			Death or ADL dependency, aOR (95% CI)^d^		
	Low	Intermediate	High	Low	Intermediate	High
Leisure time						
Model 1	1 [Reference]	0.54 (0.38-0.77)	0.47 (0.31-0.73)	1 [Reference]	0.66 (0.39-1.18)	0.34 (0.16-0.71)
Model 2	1 [Reference]	0.61 (0.42-0.88)	0.59 (0.38-0.93)	NA	NA	NA
Work time						
Model 1	1 [Reference]	0.73 (0.54-1.01)	0.92 (0.68-1.24)	1 [Reference]	0.84 (0.53-1.35)	0.79 (0.49-1.27)
Model 2	1 [Reference]	0.78 (0.57-1.07)	0.94 (0.69-1.27)	NA	NA	NA
Transport related						
Model 1	1 [Reference]	0.69 (0.52-0.93)	0.80 (0.59-1.09)	1 [Reference]	0.64 (0.40-1.01)	0.88 (0.55-1.41)
Model 2	1 [Reference]	0.73 (0.54-0.98)	0.87 (0.63-1.19)	NA	NA	NA
Household						
Model 1	1 [Reference]	1.02 (0.76-1.38)	0.91 (0.62-1.35)	1 [Reference]	1.29 (0.80-2.14)	1.14 (0.62-2.10)
Model 2	1 [Reference]	1.00 (0.74-1.35)	0.98 (0.66-1.44)	NA	NA	NA

Abbreviations: ADL, activities of daily living; aHR, adjusted hazard ratio; aOR, adjusted odds ratio; NA, not applicable.

^a^ Associations are shown with incidence of first stroke during 20-year follow-up and death or ADL dependency at 3 months after stroke.

^b^ Model 1 was adjusted for age and sex. Model 2 was adjusted for all covariates (age, sex, smoking, alcohol intake, education, economy, marital status, social network, living area, obesity, diabetes, hyperlipidemia, hypertension, atrial fibrillation, family history of stroke, apolipoprotein E ε4, insertions or deletions in Apolipoprotein C1, and consumption of sweets, vegetables, and red meat >5 times/wk).

^c^ The aHRs were calculated using Cox proportional hazard models.

^d^ The aORs were calculated using binary logistic regression models. Model 2 was not applied to death or ADL dependency to prevent overfitting given the limited number of outcome events.

Data on physical activity from 2 times were available in a subset of 1394 participants, with a median (range) time of 13.0 years (3576-5374 days) between measurements. In total, a change in activity levels was reported at follow-up by 541 of 1379 participants with data (39.2%) for leisure time physical activity, 651 of 1371 participants with data (47.5%) for work time physical activity, 721 of 1377 participants with data (52.4%) for transport physical activity, and 631 of 1378 participants with data (45.8%) for household physical activity (eFigures 2-5 in Supplement 1). In the analysis including repeated measurements, a high level of leisure time physical activity remained associated with a reduced stroke hazard (aHR, 0.46; 95% CI, 0.22-0.94). However, there were no such associations for work time, transport, or household physical activity (Table 3).

**Table 3.** Associations Between 2 Repeated Measurements of Physical Activity and Incidence of First Stroke

Physical activity level during follow-up	First stroke, aHR (95% CI) (n = 1394)^a^			
	Leisure time	Work time	Transport related	Household
Low	1 [Reference]	1 [Reference]	1 [Reference]	1 [Reference]
Intermediate	0.68 (0.37-1.23)	0.86 (0.49-1.51)	0.77 (0.48-1.23)	1.21 (0.72-2.03)
High	0.46 (0.22-0.94)	0.83 (0.48-1.44)	0.70 (0.42-1.19)	0.81 (0.41-1.59)

Abbreviation: aHR, adjusted hazard ratio.

^a^ Participants included in these analyses had available data from baseline and the reexamination. The aHRs were computed using mixed-effects Cox regression models, with physical activity level included as a time-dependent categorical variable measured at 2 times during a 20-year follow-up (2001-2004 and again at 2014-2016). All associations are adjusted for age and sex.

Exploratory subgroup analyses to identify possible interactions between leisure time physical activity and other baseline characteristics are presented in Table 4. An interaction was found between leisure time physical activity and smoking, such that smoking (current smoking or smoking in the past year vs no smoking) was associated with an increased stroke hazard in participants with low or intermediate physical activity levels (aHR, 2.33; 95% CI, 1.72-3.15) but not participants with high physical activity levels (aHR, 1.09; 95% CI, 0.63-1.87). Another interaction was found between physical activity and family history of stroke, in which having a first-degree relative with a history of stroke was associated with increased stroke hazard in participants with low or intermediate physical activity levels (aHR, 1.73; 95% CI, 1.27-2.38) but not participants with high physical activity levels (aHR, 0.79; 95% CI, 0.38-1.62).

**Table 4.** Association of Baseline Characteristics With First Stroke Incidence and Interaction With Leisure Time Physical Activity

Characteristic	Low or intermediate leisure time physical activity		High leisure time physical activity			*P* for interaction
	No. (%) of participants (n = 2635)	First stroke, aHR (95% CI)^a^		No. (%) of participants (n = 979)	First stroke, aHR (95% CI)^a^	
Smoking^b^	1423 (54.0)	2.33 (1.72-3.15)		418 (42.7)	1.09 (0.63-1.87)	.02
High alcohol intake^c^	340 (12.9)	1.08 (0.75-1.57)		127 (13.0)	1.14 (0.57-2.29)	.84
Food consumption >5 times/wk						
Sweets	1020 (38.7)	0.89 (0.68-1.18)		369 (37.7)	1.72 (0.95-2.90)	.06
Vegetables	741 (28.1)	1.06 (0.81-1.40)		290 (29.6)	0.96 (0.55-1.67)	.69
Red meat	959 (36.4)	0.88 (0.63-1.22)		418 (42.7)	1.22 (0.66-2.25)	.29
Education >12 y	708 (26.9)	0.69 (0.47-1.01)		398 (40.7)	0.84 (0.46-1.56)	.47
Financial stability^d^	2320 (88.0)	0.52 (0.33-0.82)		871 (89.0)	1.05 (0.25-4.44)	.45
Married or partnered	1966 (74.6)	0.72 (0.54-0.97)		742 (75.8)	1.09 (0.52-2.26)	.28
Social network^e^	2168 (82.3)	0.78 (0.56-1.08)		871 (89.0)	0.66 (0.30-1.46)	.74
Urban metropolitan area	1209 (45.9)	1.09 (0.84-1.43)		428 (43.7)	0.72 (0.40-1.28)	.26
Obesity	540 (20.5)	1.36 (1.02-1.80)		104 (10.6)	1.49 (0.72-3.08)	.75
Diabetes	102 (3.9)	1.63 (1.01-2.66)		26 (2.7)	2.50 (0.98-6.40)	.56
Hyperlipidemia	350 (13.3)	1.14 (0.82-1.58)		71 (7.3)	1.44 (0.71-2.93)	.63
Hypertension	483 (18.3)	1.62 (1.21-2.16)		111 (11.3)	2.26 (1.23-4.13)	.14
Atrial fibrillation	182 (6.9)	1.24 (0.76-2.03)		54 (5.5)	1.91 (0.76-4.81)	.78
Family history of stroke	444 (16.9)	1.73 (1.27-2.38)		132 (13.5)	0.79 (0.38-1.62)	.03
APOE ε4	685 (26.0)	1.01 (0.75-1.35)		254 (25.9)	1.26 (0.70-2.27)	.58
APOC1 insertion or deletion	476 (18.1)	1.54 (1.17-2.03)		118 (12.1)	1.45 (0.79-2.66)	.65

Abbreviations: aHR, adjusted hazard ratio; APOC1, apolipoprotein C1 gene; APOE ε4, apolipoprotein E gene ε4 allele.

^a^ Incidence was measured during a 20-year follow-up, and aHRs for baseline characteristics were computed for low or intermediate and high leisure time physical activity using Cox proportional hazard regression. All associations are adjusted for age and sex.

^b^ Smoking was defined as current smoking or smoking in the past year.

^c^ High alcohol intake was defined as more than 10 g/d for females and more than 20 g/d for males.

^d^ Financial stability was defined as managing monthly expenses without difficulty.

^e^ Presence of a social network was defined as communication or engagement with 3 or more familiar individuals per week.

## Discussion

In this 20-year prospective, population-based cohort study, we observed that intermediate (eg, bicycling, walking outdoors, or playing table tennis ≥4 hr/wk) and high (eg, running, swimming, or playing tennis 2-3 hr/wk or engaging in competitive sports) levels of leisure time physical activity were associated with a reduction in stroke incidence. Similar benefits were found for an intermediate physical activity level in transportation (eg, walking or biking 20-40 min/d). A high level of leisure time physical activity was also associated with a lower risk of poststroke death or ADL dependency, and there was a potentially mitigating interaction between physical activity and the stroke risk associated with smoking or having a family history of stroke.

Consistent with our findings, leisure time physical activity has been associated with a decreased risk of stroke in several studies.^10,11,12^ In contrast, some studies have found that high levels of work time physical activity were associated with increased risk of stroke^14^ and overall cardiovascular disease and mortality,^24,25^ whereas other studies^13,26^ found a decreased stroke incidence with increased work time physical activity. Few prior studies have investigated the association of transport or household physical activity with stroke. However, a 2022 retrospective study^26^ on the US population found that individuals who walked, bicycled, or did house or yard work had a significant reduction in stroke prevalence. Daily walking or cycling to and from work has also been shown to be associated with a reduced risk of stroke.^11^

Emerging evidence suggests that prestroke physical activity is associated with improved stroke outcomes.^8^ However, most prior studies have used retrospective assessments of prestroke physical activity, limiting the interpretability of their findings. Using prospective assessments, Mediano et al^27^ reported that higher levels of total, leisure time, and work time physical activity were associated with a lower risk of mortality. In addition, prestroke physical activity has been associated with decreased poststroke mortality in several studies using retrospective assessments.^28,29,30,31^ Similar to our results, those of Rist et al^32^ demonstrated that increased levels of prestroke physical activity were associated with greater independence in instrumental ADL tasks assessed 3 years after a stroke.

Self-reported assessments of physical activity typically exhibit limited association with objective data,^33^ with the latter being more consistently associated with cardiovascular outcomes.^34^ Leisure time and transport physical activity were positively correlated with pedometer-derived measurements in this study, whereas work time and household activities were not. Considering that leisure time and transport physical activities were also the only domains associated with stroke incidence, aerobic activities (ie, a higher daily step count) may be particularly beneficial for stroke prevention.^18,19^ However, observed correlations were weak. Given that pedometers measured total physical activity over a few days while questionnaires measured domain-specific physical activity over a year, discrepancies were expected. Still, the absence of stronger correlation suggests that there is limited validity in capturing the true physical activity level through domain-specific questions used in our investigation. Some physical activity behaviors may also exhibit less stability over time, whereas self-reported leisure time physical activity may represent a consistent, often long-term behavior.^35^ In support of this conclusion, leisure time physical activity exhibited the smallest intraindividual change over time.

### Strengths and Limitations

Our findings should be interpreted considering several strengths and limitations. Strengths include the prospective population-based design, a long-term follow-up, and robust measures of stroke incidence, mortality, and poststroke ADL dependency through extensive data collection. The possibility to link the INTERGENE cohort with data from the Swedish national registers is a particular strength of this study. We were also able to adjust for a wide range of important risk factors and potential confounders, for which data were not available in prior studies. Furthermore, our study benefited from the high validity and completeness of Swedish register data, minimizing data quality concerns and missing observations.^36,37^

Limitations include the study participation rate of 41.9% at baseline, which may have introduced participation bias. Additionally, most participants were assessed only once, which limits our understanding of longitudinal changes in physical activity behaviors. Second, self-reported measures of physical activity are susceptible to social desirability and recall bias.^15^ Physical activity assessments referred to the year mean, which may increase the uncertainty of estimates. However, 1-year estimates ensure a consistent recall period across participants without effects from seasonal variations. Furthermore, the objective evaluation of physical activity was conducted in a subset of participants, and the representativeness of pedometer data in reflecting usual physical activity levels is limited. Although Swedish government registers have excellent coverage, they do not capture stroke events or deaths if a person has emigrated, and we were not able to control for this factor. Additionally, despite adjustment for multiple baseline characteristics, the potential for unmeasured confounding and the influence of time-varying factors cannot be ruled out. The observational design of our study also renders it susceptible to reverse causality, wherein individuals with specific health conditions may have altered their physical activity levels due to their health status.

## Conclusions

In this cohort study of 3614 individuals from the general population in western Sweden, intermediate and high levels of leisure time physical activity and an intermediate level of transport physical activity were associated with a decreased risk of stroke. Higher levels of leisure time physical activity at baseline were also associated with a lower risk of death or ADL dependency 3 months after stroke. These findings suggest that integrating leisure time physical activity and active transport into public health policies may be a sustainable intervention to reduce the burden of stroke.

## Notes

Supplement 1. **eTable 1.** Classification of physical activity domains at baseline in 2001 to 2004 and at the reexamination in 2014 to 2016 with pedometer-derived data **eTable 2.** Adjusted associations between levels of baseline domain-specific physical activity with the incidence of first stroke during a 20-year follow-up and with death or ADL dependency at 3 months after stroke **eFigure 1.** Crude cumulative incidence of first stroke during a 20-year follow-up stratified by domain-specific physical activity **eFigure 2.** Intraindividual change in leisure time physical activity between baseline at 2001 to 2004 and the reexamination 2014 to 2016 in 1379 participants with available data **eFigure 3.** Intraindividual change in work time physical activity between baseline at 2001 to 2004 and the reexamination 2014 to 2016 in 1371 participants with available data **eFigure 4.** Intraindividual change in transport physical activity between baseline at 2001 to 2004 and the reexamination 2014 to 2016 in 1377 participants with available data **eFigure 5.** Intraindividual change in household physical activity between baseline at 2001 to 2004 and the reexamination 2014 to 2016 in 1378 participants with available data

Supplement 2.
Data Sharing Statement
