# Supplementary material for: Domain-Specific Physical Activity and Stroke in Sweden
Source: JAMA Netw Open. 2024 May 29;7(5):e2413453. doi: 10.1001/jamanetworkopen.2024.13453 (PMC11137634; doi:10.1001/jamanetworkopen.2024.13453)
Supplement: Supplement 1. — eTable 1. Classification of physical activity domains at baseline in 2001 to 2004 and at the reexamination in 2014 to 2016 with pedometer-derived data eTable 2. Adjusted associations between levels of baseline domain-specific physical activity with the incidence of first stroke during a 20-year follow-up and with death or ADL dependency at 3 months after stroke eFigure 1. Crude cumulative incidence of first stroke during a 20-year follow-up stratified by domain-specific physical activity eFigure 2. Intraindividual change in leisure time physical activity between baseline at 2001 to 2004 and the reexamination 2014 to 2016 in 1379 participants with available data eFigure 3. Intraindividual change in work time physical activity between baseline at 2001 to 2004 and the reexamination 2014 to 2016 in 1371 participants with available data eFigure 4. Intraindividual change in transport physical activity between baseline at 2001 to 2004 and the reexamination 2014 to 2016 in 1377 participants with available data eFigure 5. Intraindividual change in household physical activity between baseline at 2001 to 2004 and the reexamination 2014 to 2016 in 1378 participants with available data [file jamanetwopen-e2413453-s001.pdf]

Supplemental Online Content

Viktorisson A, Palstam A, Nyberg F, Berg C, Lissner L, Sunnerhagen KS. Domain-specific physical activity and stroke in Sweden. *JAMA Netw Open*. 2024;7(5):e2413453. doi:10.1001/jamanetworkopen.2024.13453

eTable 1. Classification of physical activity domains at baseline in 2001 to 2004 and at the reexamination in 2014 to 2016 with pedometer-derived data

eTable 2. Adjusted associations between levels of baseline domain-specific physical activity with the incidence of first stroke during a 20-year follow-up and with death or ADL dependency at 3 months after stroke

eTable 1. Classification of physical activity domains at baseline in 2001 to 2004 and at the reexamination in 2014 to 2016 with pedometer-derived data

eTable 2. Adjusted associations between levels of baseline domain-specific physical activity with the incidence of first stroke during a 20-year follow-up and with death or ADL dependency at 3 months after stroke

eFigure 1. Crude cumulative incidence of first stroke during a 20-year follow-up stratified by domain-specific physical activity

eFigure 2. Intraindividual change in leisure time physical activity between baseline at 2001 to 2004 and the reexamination 2014 to 2016 in 1379 participants with available data

eFigure 3. Intraindividual change in work time physical activity between baseline at 2001 to 2004 and the reexamination 2014 to 2016 in 1371 participants with available data

eFigure 4. Intraindividual change in transport physical activity between baseline at 2001 to 2004 and the reexamination 2014 to 2016 in 1377 participants with available data

eFigure 5. Intraindividual change in household physical activity between baseline at 2001 to 2004 and the reexamination 2014 to 2016 in 1378 participants with available data

This supplemental material has been provided by the authors to give readers additional information about their work.

© 2024 Viktorisson A et al. *JAMA Network Open.*

eTable 1. Classification of physical activity domains at baseline in 2001 to 2004, and at the re-examination in 2014 to 2016 with pedometer-derived data.

		**Number of participants**			**Number of steps per day,** Median (Q_1, Q_3)
		**Baseline 2001-2004** (n=3 614)	**Re-examination 2014-2016** (n=1 394)	**Pedometer data available** n=496)	
**Leisure-time physical activity**					
Low	Almost completely inactive, reading, watching television, watching movies, or using the computer.	n=370	n=148	n=27	5614 (3788-8755)
Intermediate	Activities such as riding a bicycle, walking outdoors, table tennis ≥4 hours/week.	n=2264	n=822	n=298	8027 (6138-10338)
High	Activities such as running, swimming, playing tennis, badminton, calisthenics 2-3 hours/week.	n=885	n=381	n=167	9355 (6961-12325)
	Hard physical training for competitive sports several times per week.	n=71	n=28		
**Work-time physical activity**					
Low	Mostly sedentary or being unemployed*	n=1078	n=475	n=149	7838 (6458-10611)
Intermediate	Passive sitting approximately half of the time	n=981	n=398	n=197	8489 (6180-11184)
	Mostly standing	n=271	n=97		
High	Mostly walking / lifting, minor carrying	n=827	n=312	n=144	8412 (6198-10539)
	Mostly walking / lifting, major carrying	n=318	n=70		
	Heavy manual labor	n=111	n=19		
**Transport-related physical activity**					
Low	Transportation by walking or biking almost never	n=341	n=117	n=107	7512 (4825-10081)
	Transportation by walking or biking < 20 min/day	n=853	n=290		
Intermediate	Transportation by walking or biking 20-40 min/day	n=1499	n=564	n=218	8294 (6439-10300)
High	Transportation by walking or biking 40-60 min/day	n=535	n=251	n=168	9007 (6787-11851)
	Transportation by walking or biking 60-90 min/day	n=344	n=155		
**Household physical activity**					
Low	Household work for 0 hours/day	n=1113	n=414	n=133	8580 (6617-11085)
Intermediate	Household work for 1-2 hours/day	n=1636	n=690	n=267	8516 (6548-11056)
High	Household work for 3-4 hours/day	n=667	n=229	n=91	7124 (4923-10229)
	Household work for 5-6 hours/day	n=118	n=38		
	Household work for 7-8 hours/day	n=31	n=5		
	Household work for >8 hours/day	n=18	n=2		

The mean number of steps per day were recorded using a sealed pedometer over a period of six days. Leisure-time physical activity was determined using a modified version of the Saltin-Grimby physical activity level scale. Q_1= first quartile Q_3= third quartile * n=121 participants were categorized as having low work-time physical activity due to being unemployed.

© 2024 Viktorisson A et al. *JAMA Network Open.*

**eTable 2.** Adjusted associations between levels of baseline domain-specific physical activity with the incidence of first stroke during a 20-year follow-up and with death or ADL dependency at 3 months after stroke.						
	Stroke incidence<br>Adjusted hazard ratio (95% confidence interval)			Death or ADL dependency<br>Adjusted odds ratio (95% confidence interval)		
Leisure-time physical activity	Low	Intermediate	High	Low	Intermediate	High
Model 1 (lifestyle factors)	1 [Reference]	0.57 (0.40-0.83)	0.53 (0.35-0.83)	1 [Reference]	0.69 (0.39-1.20)	0.37 (0.18-0.79)
Model 2 (socioeconomic factors)	1 [Reference]	0.58 (0.40-0.83)	0.53 (0.35-0.83)	1 [Reference]	0.75 (0.42-1.31)	0.41 (0.19-0.88)
Model 3 (comorbid conditions)	1 [Reference]	0.59 (0.41-0.86)	0.54 (0.35-0.84)	1 [Reference]	0.70 (0.40-1.23)	0.37 (0.17-0.78)
Model 4 (genetic factors)	1 [Reference]	0.53 (0.37-0.76)	0.47 (0.31-0.72)	1 [Reference]	0.66 (0.38-1.15)	0.34 (0.16-0.72)
Work-time physical activity						
Model 1 (lifestyle factors)	1 [Reference]	0.76 (0.55-1.04)	0.93 (0.69-1.26)	1 [Reference]	0.88 (0.55-1.41)	0.83 (0.52-1.33)
Model 2 (socioeconomic factors)	1 [Reference]	0.74 (0.54-1.01)	0.90 (0.67-1.22)	1 [Reference]	0.88 (0.54-1.40)	0.81 (0.50-1.30)
Model 3 (comorbid conditions)	1 [Reference]	0.77 (0.56-1.05)	0.95 (0.70-1.28)	1 [Reference]	0.86 (0.54-1.37)	0.81 (0.51-1.30)
Model 4 (genetic factors)	1 [Reference]	0.74 (0.54-1.01)	0.92 (0.68-1-24)	1 [Reference]	0.84 (0.53-1.35)	0.78 (0.49-1.25)
Transport-related physical activity						
Model 1 (lifestyle factors)	1 [Reference]	0.70 (0.52-0.94)	0.85 (0.62-1.16)	1 [Reference]	0.63 (0.40-1.01)	0.89 (0.55-1.43)
Model 2 (socioeconomic factors)	1 [Reference]	0.72 (0.54-0.97)	0.83 (0.61-1.13)	1 [Reference]	0.69 (0.43-1.10)	0.95 (0.59-1.53)
Model 3 (comorbid conditions)	1 [Reference]	0.72 (0.53-0.96)	0.85 (0.62-1.16)	1 [Reference]	0.65 (0.41-1.04)	0.93 (0.58-1.49)
Model 4 (genetic factors)	1 [Reference]	0.68 (0.51-0.92)	0.79 (0.58-1.07)	1 [Reference]	0.64 (0.40-1.01)	0.87 (0.55-1.40)
Household physical activity						
Model 1 (lifestyle factors)	1 [Reference]	0.98 (0.72-1.32)	0.95 (0.64-1.40)	1 [Reference]	1.27 (0.77-2.09)	1.22 (0.67-2.24)
Model 2 (socioeconomic factors)	1 [Reference]	1.04 (0.77-1.40)	0.97 (0.65-1.43)	1 [Reference]	1.36 (0.83-2.24)	1.30 (0.71-2.40)
Model 3 (comorbid conditions)	1 [Reference]	1.01 (0.74-1.35)	0.90 (0.61-1.33)	1 [Reference]	1.28 (0.78-2.11)	1.13 (0.61-2.08)
Model 4 (genetic factors)	1 [Reference]	1.03 (0.76-1.38)	0.89 (0.60-1.32)	1 [Reference]	1.29 (0.79-2.12)	1.14 (0.62-2.10)

Adjusted hazard ratios were calculated using Cox proportional hazard models. Adjusted odds ratios were calculated using binary logistic regression models. Model 1 was adjusted for lifestyle factors (smoking, alcohol intake, and consumption of sweets, vegetables and red meat > 5 times/week); Model 2 was adjusted for socioeconomic factors (education, economy, marital status, social network, and living area); Model 3 was adjusted for comorbid conditions (obesity, diabetes mellitus, hyperlipidemia, hypertension, and atrial fibrillation); and Model 4 was adjusted for genetic factors (family history of stroke, Apolipoprotein E ε4, and insertions or deletions in Apolipoprotein C1). All associations are also adjusted for age and sex.

© 2024 Viktorisson A et al. *JAMA Network Open.*

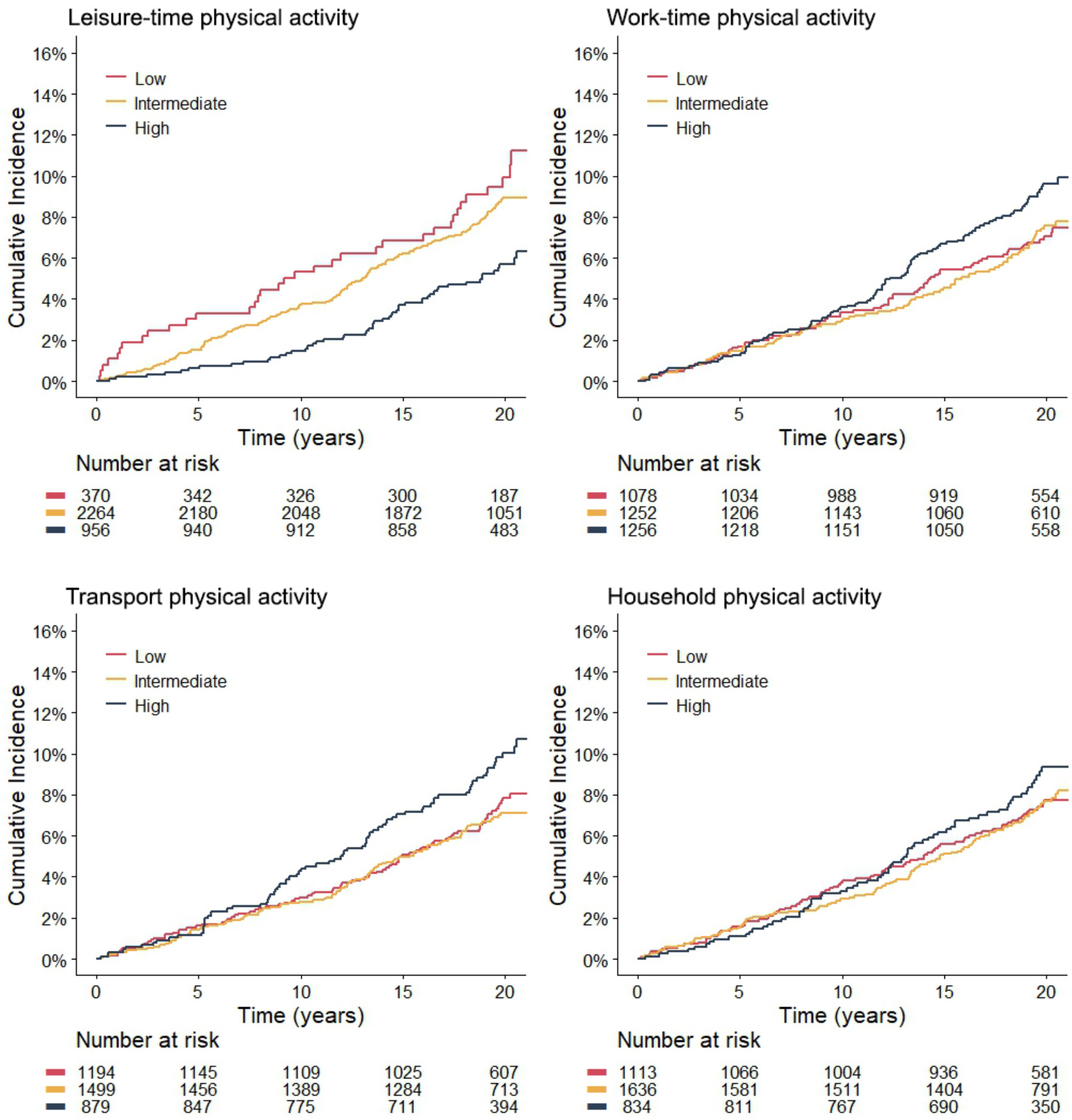

eFigure 1. Crude cumulative incidence of first stroke during a 20-year follow-up stratified by domain-specific physical activity.

© 2024 Viktorisson A et al. *JAMA Network Open.*

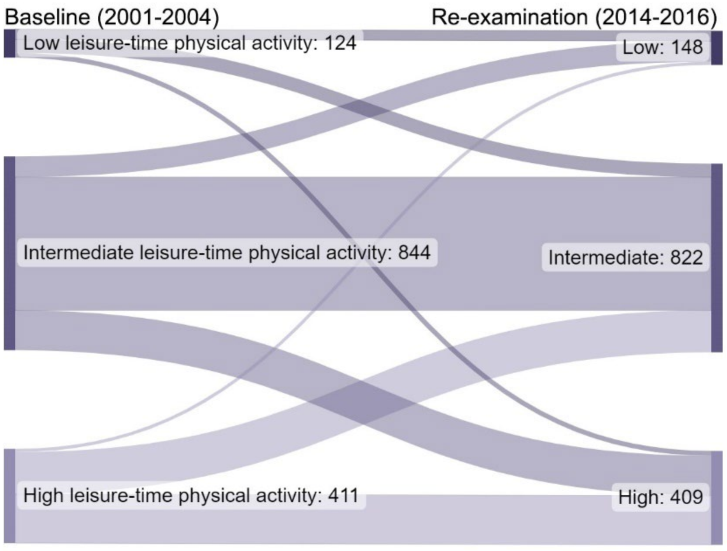

eFigure 2. Intraindividual change in leisure-time physical activity between baseline at 2001 to 2004, and the re-examination 2014 to 2016 in 1379 participants with available data.

© 2024 Viktorisson A et al. *JAMA Network Open.*

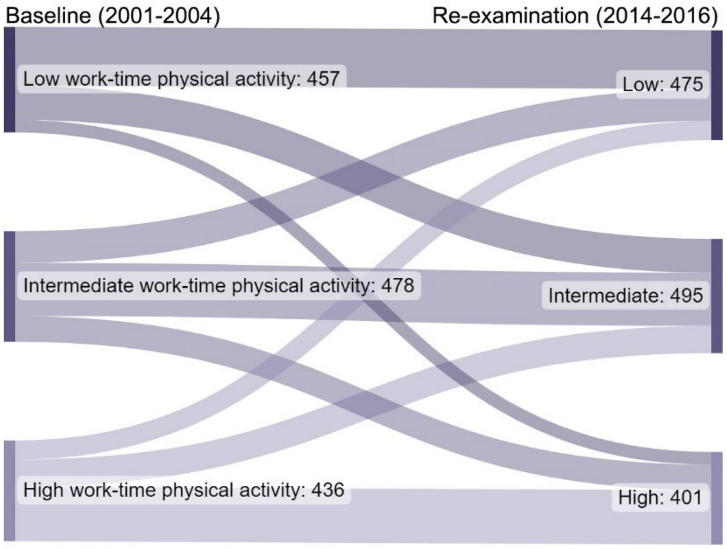

eFigure 3. Intraindividual change in work-time physical activity between baseline at 2001 to 2004, and the re-examination 2014 to 2016 in 1371 participants with available data.

© 2024 Viktorisson A et al. *JAMA Network Open.*

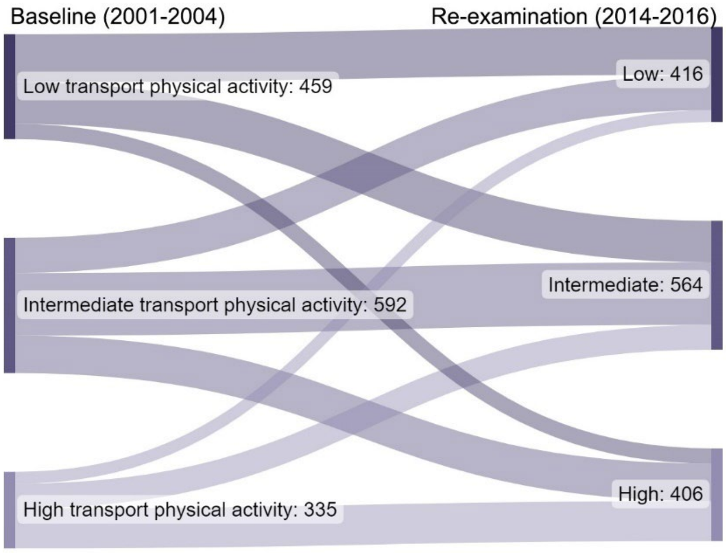

eFigure 4. Intraindividual change in transport physical activity between baseline at 2001 to 2004, and the re-examination 2014 to 2016 in 1377 participants with available data.

© 2024 Viktorisson A et al. *JAMA Network Open.*

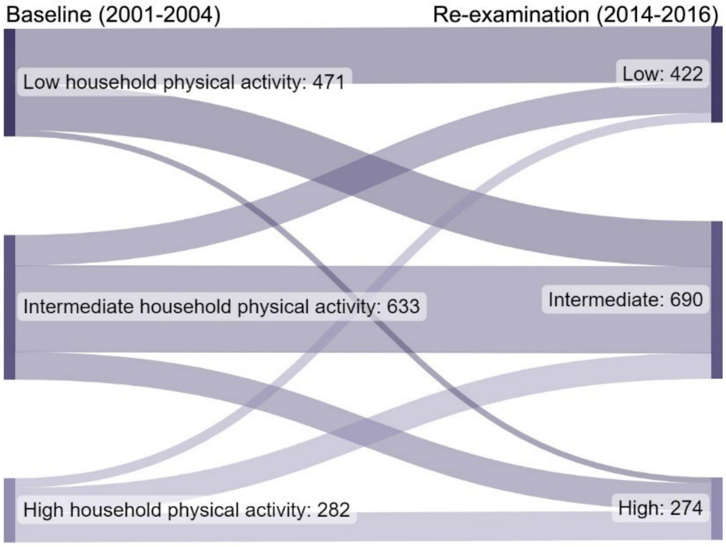

eFigure 5. Intraindividual change in household physical activity between baseline at 2001 to 2004, and the re-examination 2014 to 2016 in 1378 participants with available data.

© 2024 Viktorisson A et al. *JAMA Network Open.*
