# Supplementary material for: Domain-Specific Physical Activity and Stroke in Sweden
Source: JAMA Netw Open. 2024 May 29;7(5):e2413453. doi: 10.1001/jamanetworkopen.2024.13453 (PMC11137634; doi:10.1001/jamanetworkopen.2024.13453)
Supplement: Supplement 2. — Data Sharing Statement [file jamanetwopen-e2413453-s002.pdf]

Data Sharing Statement

Viktorisson. Domain-Specific Physical Activity and Stroke in Sweden. *JAMA Netw Open*. Published online May 29, 2024. doi:10.1001/jamanetworkopen.2024.13453

Data
Data available: No

Additional Information
Explanation for why data not available: Data supporting our findings can be obtained by contacting the corresponding author, on behalf of the INTERGENE steering group, after a review of the secrecy. According to Swedish regulations (epn.se/en/start/regulations/), permission to use data can only be based on application and approval from the Swedish Ethical Review Authority.
